# Incidence of Primary Central Nervous System Tumors Among the Hispanic Population in Texas 1995-2013

**DOI:** 10.7759/cureus.10235

**Published:** 2020-09-03

**Authors:** Solomon Ambe, Damir Nizamutdinov, Jason H Huang, Ekokobe Fonkem

**Affiliations:** 1 Neurology, Tulane University School of Medicine, New Orleans, USA; 2 Neurosurgery, Baylor Scott & White Health, Temple, USA; 3 Neurology, Barrow Neurological Institute, Dignity Health, Phoenix, USA

**Keywords:** central nervous system tumors, epidemiology, texas, geographic variation

## Abstract

Introduction: Primary central nervous system (CNS) tumors constitute a group of rare and heterogeneous tumors. A rising incidence rate in the United States has been linked to modern changes in early diagnosis and reporting. This study aims to examine temporal incidence trends, geographic variation, and the average annual age-adjusted rates among Hispanic populations in Texas from 1995 to 2013.

Methods: SEER*STAT (Surveillance Research Program, National Cancer Institute (seer.cancer.gov/seerstat) version 8.3.2) and Joinpoint Regression 4.4.0.0 (Statistical Methodology and Applications Branch, Surveillance Research Program, National Cancer Institute) software were used to analyze incidence of primary brain and CNS tumors among Texas residents. Data were obtained from the Texas Cancer Registry of the Texas Department of State Health Services.

Results: From the 30,122 cases of primary CNS tumors diagnosed throughout the state of Texas from 2008 to 2012, the overall average annual age-adjusted incidence rate for Hispanics and non-Hispanics combined was 25.35 per 100,000 persons. Among Hispanics, West Texas had the highest incidence trends and the highest average age-adjusted incidence rate of 27.17, followed by North Texas at 26.01 and the Panhandle at 23.63. East Texas had the lowest incidence rate of 16.23. The incidence trend among Hispanics has decreased consistently at a rate of 0.83 % from 1995 to 2013.

Conclusions: The incidence of tumors was more pronounced in the Hispanic population in northern Texas compared with southern Texas. The presence of oil and gas production industries with farming and construction could play a role in the observed incidence of disease. Further studies with a focus on occupational health among Hispanics in Texas will be needed to elucidate the cause of such distribution.

## Introduction

Primary central nervous system (CNS) tumors are tumors that originate from the cells in the CNS. Secondary CNS tumors start from other parts of the body and spread to the brain and/or CNS. The incidence of primary CNS tumors in the United States is 21.97 per 100,000 people, with higher incidence in women than men [1]. Compared with populations across the globe, the incidence rate of primary brain tumors is much higher in the U.S. at 7.2 vs 3.4 cases per 100,000 people. Moreover, incidence is generally higher in developed nations compared with developing nations at 5.1 vs 3.0 per 100,000 people [1,2].

Additionally, a recent study on the worldwide patterns and trends on the incidence of CNS tumors revealed a five-fold difference between the highest rates in Europe compared with the lowest rates in Asia; meanwhile, South America revealed a pattern of incidences that continue to increase [3].

In the U.S., the Central Brain Tumor Registry of the United States (CBTRUS) produces the statistical reports on all primary CNS tumors based on data collected from the Center for Disease Control and Prevention, the National Program for Cancer Registry, and the National Cancer Institute, Surveillance, Epidemiology and End Results (SEER) program [4]. 

Although national statistics exist, it doesn’t appear that any significant studies have been performed on the geographical variation in incidence and temporal trends from states with high Hispanic populations (eg, California, New Mexico, Arizona, Texas). Due to insufficient findings from research on racial and ethnic health disparities [5], a two-decade retrospective study on incidence and geographic variation of CNS tumors of the Hispanic population is warranted.

The objective of this is study is to examine trends in incidence of primary CNS tumors, including the rest of craniospinal neuraxis, among Hispanics vs non-Hispanics in the state of Texas from 1995 to 2013. Additionally, the study aims to provide a statewide status report on geographic variation and temporal trends in the incident rate of CNS tumors that occur among Hispanics in Texas. Categorizing the incidence by geographic region and socio-demographic characteristics would provide salient differences in the trends and rates of distribution of tumors. Furthermore, this will assist in revealing any peculiarities from different geographic regions within the same subpopulation.

## Materials and methods

Ethics

This study meets the National Institute of Health (NIH) and Baylor Scott & White Health Care Central Texas Institutional Review Board (IRB) guidelines. All human investigations were performed after approval by an IRB and in accordance with an assurance filed with and approved by the U.S. Department of Health and Human Services.

Data source

Data was obtained from the Texas Cancer Registry, Cancer Epidemiology and Surveillance Branch of the Texas Department of State Health Services. The dataset included incidence of primary CNS tumors among Texas residents from 1995 to 2013. Individual-level data for non-residents and individual-level data from the U.S. Department of Veteran Affairs were not included [6]. The current population data of the geographic regions of Texas were obtained online from the SEER website. The data analysis included all primary CNS tumors of all age groups and behavior.

Statistics 

SEER*Statstatistical software (Surveillance Research Program, National Cancer Institute (seer.cancer.gov/seerstat) version 8.3.2) was used to calculate the counts, age-adjusted incidence rates, frequencies, and other relevant statistics for the years 2008 to 2012 for both malignant and non-malignant tumors. Age-adjustment for all primary CNS tumors was standardized to the 2000 U.S. Census Standard population-based on five-year age groupings. Age-specific incidence rates by five-year age groups were also calculated per 100,000 (the total frequencies may not add up to 100% due to rounding).

Joinpoint Regression (Statistical Methodology and Applications Branch, Surveillance Research Program, National Cancer Institute, version 4.4.0.0) analysis was done for the incidence trends from 1995 to 2013 using only malignant tumors because non-malignant tumors were not collected until after 2002 following the passage of the Benign Tumor Act by congress mandating data collection of non-malignant brain tumors [7].

The statistics by ICD-O-3 (defined by World Health Organization (WHO)) primary tumors’ sites used by Central Brain Tumor Registry of the United States (CBTRUS) in 2015 are grouped as follows: nasal cavity (C30.0) cerebral meninges (C70.0); spinal meninges (C70.1); meninges (C70.9); cerebrum (C71.0); frontal lobe (C71.1); temporal lobe (C71.2); parietal lobe (C71.3); occipital lobe (C71.4); ventricle (C71.5); cerebellum (C71.6); brain stem (C71.7); overlapping lesion of the brain (C71.8); brain (C71.9); spinal cord (C72.0); cauda equine (C72.1); olfactory nerve (C72.2); optic nerve (C72.3); acoustic nerve (C72.4); cranial nerve (C72.5); overlapping lesion of brain and CNS (C72.8); nervous system (C72.9); pituitary gland (C75.1); craniopharyngeal duct (C75.2); and pineal gland (C75.3).

Other software/sources

Microsoft Office Excel and Word programs were used to generate the graphs and tables. Race was classified as binary: Hispanics and non-Hispanics. The geographic regions of Texas were grouped into seven regions following the classification of the website www.texas counties.net for data analysis.

## Results

Incidence rates by race and gender

A total of 30,122 primary CNS tumors were diagnosed in the State of Texas from 2008 to 2012. The overall annual age-adjusted incidence rate was 25.35 per 100,000 population (95% CI: 25.06-25.64). Females had a higher rate compared with males (28.31 vs 22.1). Hispanics have a lower annual age-adjusted incidence rate compared with non-Hispanics (22.61 with a 95% CI 22.06-23.17 vs 26.69 with a 95% CI 26.34-27.05). Among Hispanics, females have a higher incidence rate compared with males (25.72 vs 19.27), likewise among the non-Hispanic population (29.53 vs 23.53).

Incidence by behavior

The annual age-adjusted incidence rate of malignant tumor was 36% higher in non-Hispanics compared with Hispanics (8.06 vs 5.93 per 100,000 population). For malignant tumors, the age-adjusted incidence rates are higher in males than females in both Hispanics (6.95 vs 5.09) and non-Hispanics (9.50 vs 6.77). For non-malignant tumors females have a higher incidence rate for both Hispanics and non-Hispanics (18.89 and 20.92, respectively) compared with male Hispanics and non-Hispanics (10.49 and 12.14, respectively).

Incidence trends 1995 to 2013

**Figure 1:**
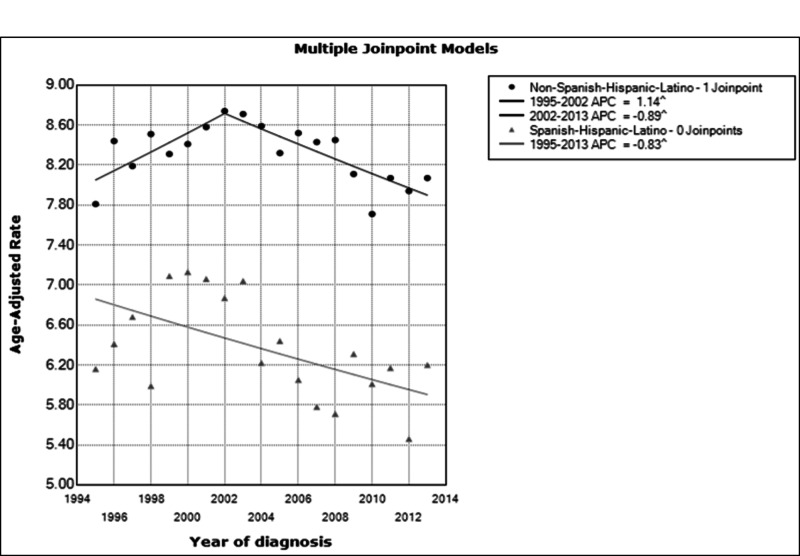
Graph showing incidence trend of malignant primary CNS tumors in Texas among Hispanics and non-Hispanics 1995-2013. APC: annual percent change, CNS: central nervous system

The incidence trends for malignant tumors from year 1995 to 2013 were remarkably different for Hispanic and non-Hispanic populations. The age-adjusted incidence rate for malignant primary CNS tumors among the Hispanic populations was on the decline at a rate of 0.83% per year from 1995 to 2013 (constant trend): annual percent change (APC) is -0.83 (95% CI: -1.4,-0.2; p<0.05). However, non-Hispanics have a relatively stable trend of age-adjusted rate increasing at 1.14% per year from 1995 to 2002 (APC=1.14, 95% CI 0.2, 2.4, p<0.05) (Figure 1).

From year 2002, a significant change in trend was observed and the age-adjusted incidence rate started declining at 0.9% per year through 2013 (APC=-0.9, 95% CI: -1.3, -0.4, p<0.05).

When stratified by gender, among Hispanics from 1995 to 2013, the trend in males constantly decreased at a rate of 1.05% per year (APC =-1.05, 95% CI: -0.2,-2.6; p<0.05), meanwhile the trend in annual percent change in females was not statistically significant (APC=-0.5, 95% C.I: -1.4, 0.3, p=0.2).

For non-Hispanics, males’ annual percent change was constant between 1995 and 2013 (APC=-0.11, 95% CI: -0.5, 0.3, p=0.5). Females’ annual percent change also was constant until 2004; after this period, the trend in annual percent change declined (APC=-1.36, 95% CI: -2.0, -0.7, p<0.05). Prior to 2002, the annual percent change for the total population of Texas was not statistically significant. From 2002 to 2013, there was a decrease in trend at a rate of 1.12% per year (APC=-1.12, 95% CI: -1.6,-0.7, p<0.05). The trend was the same for males and females. When stratified by geographic regions, only the Hispanic population in Central Texas revealed a statistically significant APC of -1.9 (95% CI: -3.6, -0.1, p<0.05). Ethnicity and genders are effect modifiers as they affect the trend differently.

Regional variations

**Figure 2:**
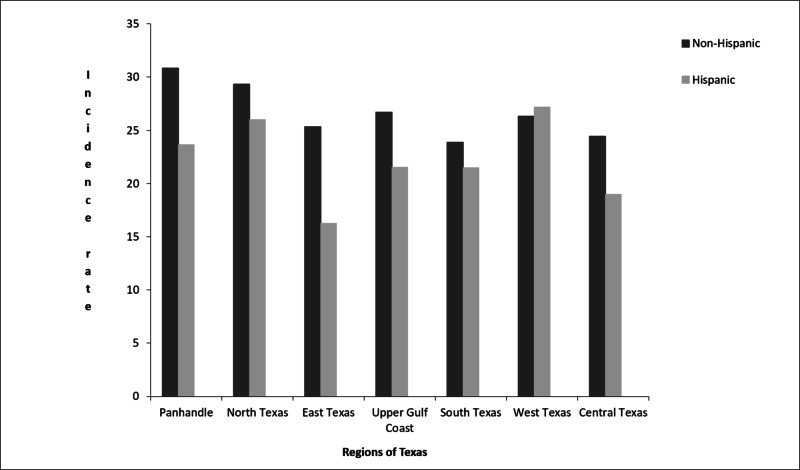
The average annual age-adjusted incidence rates per 100000 population of primary CNS tumors among Hispanics and non-Hispanics in Texas by geographic regions (2008-2012). CNS: central nervous system

The annual age-adjusted incidence rate was consistently higher in non-Hispanics than Hispanics, except in West Texas where the rate was higher in Hispanics than non-Hispanics (Figure 2).

Among Hispanics, West Texas had the highest average annual age-adjusted incidences (27.17) of primary CNS tumors from 2008-2012, followed by North Texas (26.01) and the Panhandle (23.63) (Table 1 and Figure 2).

**Table 1:** The average annual age-adjusted incidence rate 2008 to 2012 of primary CNS tumors by ICD-03/WHO 2008 morphology among Hispanics in Texas by geographic regions. CNS: central nervous system, WHO: World Health Organization

Geographic distribution	The Panhandle		North Texas		East Texas		Upper Gulf		South Texas		West Texas		Central Texas	
Race	Non-Hisp	Hisp	Non-Hisp	Hisp	Non-Hisp	Hisp	Non-Hisp	Hisp	Non-Hisp	Hisp	Non-Hisp	Hisp	Non-Hisp	Hisp
Rate	Rate 95% CI	Rate 95% CI	Rate 95% CI	Rate 95% CI	Rate 95% CI	Rate 95% CI	Rate 95% CI	Rate 95% CI	Rate 95% CI	Rate 95% CI	Rate 95% CI	Rate 95% CI	Rate 95% CI	Rate 95% CI
All brain tumors	30.8 28.2-33.7	23.6 18.2-30.0	29.3 28.7-30.0	26.0 24.3-27.8	25.4 24.3-26.4	16.2 13.0-20.1	26.7 26.0-27.5	21.5 20.2-22.9	23.9 22.8-25.0	21.5 20.6-22.4	26.3 25.0-27.7	27.2 25.7-28.8	24.5 23.5-25.4	18.9 17.1-21.1
II Lymphomas and reticuloendothelial neoplasms	0.36 0.13-0.82	0	0.5 0.41-0.59	0.4 0.23-0.63	0.4 0.28-0.55	0.25 0.03-1.1	0.51 0.42-0.63	0.44 0.28-0.66	0.31 0.20-0.46	0.61 0.47-0.79	0.23 0.13-0.39	0.61 0.40-0.89	0.43 0.31-0.57	0.47 0.21-0.89
III CNS and misc intracranial and intraspinal neoplasms	25.9 23.5-28.5	21.7 16.4-28.0	24.2 23.6-24.8	21.8 20.2-23.4	20.8 19.9-21.7	13.4 10.4-17	22.1 21.4-22.7	18.3 17.1-19.6	20.0 19.0-21.0	17.9 17.1-18.7	21.3 20.1-22.6	22.1 20.7-23.5	19.7 18.9-20.6	15.3 13.6-17.2
III(a) Ependymomas and choroid plexus tumor	0.43 0.15-0.94	0	0.61 0.51-0.71	0.37 0.21-0.6	0.48 0.34-0.65	0.05 0-0.76	0.45 0.36-0.55	0.34 0.22-0.51	0.55 0.38-.77	0.36 0.27-0.49	0.49 0.31-0.72	0.55 0.37-0.79	0.71 0.55-0.89	0.61 0.32-1.0
III(a.1) Ependymomas	0.43 0.15-0.94	0	0.53 0.44-0.62	0.35 0.19-0.58	0.42 0.29-0.59	0.05 0-0.76	0.42 0.33-0.52	0.32 0.21-0.49	0.46 0.3-0.66	0.33 0.24-0.45	0.44 0.27-0.66	0.52 0.35-0.76	0.64 0.5-0.82	0.57 0.29-1.0
III(b) Astrocytomas	5.07 4.0-6.3	2.91 1.4-5.3	4.5 4.2-4.8	3.4 2.8-4.1	4.4 4.0-4.9	2.7 1.7-4.3	4.8 4.5-5.1	3.4 2.9-3.9	5.02 4.5-5.6	3.3 3.0-3.7	4.7 4.2-5.3	3.4 3.0-4.0	5.3 4.9-5.7	2.6 1.9-3.4
III(c) Intracranial and intraspinal embryonal tumors	0.25 0.06-0.66	0.14 0-1.3	0.22 0.16-0.29	0.29 0.2-0.43	0.22 0.13-0.36	0.39 0.15-1.14	0.25 0.18-0.34	0.28 0.19-0.4	0.32 0.19-0.51	0.22 0.16-0.3	0.28 0.14-0.49	0.31 0.19-0.49	0.23 0.14-0.34	0.23 0.12-0.45
III(d) Other gliomas	0.82 0.41-1.45	0.1 0-1.19	1.28 1.15-1.43	0.71 0.5-1.99	0.8 0.61-1.02	0.61 0.1-1.9	0.92 0.78-1.06	0.57 0.43-0.74	1.2 0.9-1.5	0.6 0.47-0.76	1.1 0.81-1.5	1.0 0.76-1.3	1.0 0.82-1.2	0.77 0.43-1.3
III(d.1) Oligodendrogliomas	0.46 0.17-1.0	0	0.39 0.32-0.47	0.17 0.1-0.32	0.24 0.14-0.38	0.07 0-0.79	0.3 0.23-0.38	0.23 0.14-0.35	0.37 0.23-0.55	0.16 0.1-0.24	0.37 0.21-0.6	0.36 0.21-0.57	0.37 0.26-0.5	0.35 0.18-0.65
III(d.2) Mixed and unspecified gliomas	0.36 0.12-0.81	0.09 0-1.19	0.86 0.75-0.99	0.54 0.35-0.8	0.54 0.39-0.73	0.16 0.03-0.88	0.57 0.47-0.69	0.32 0.22-0.46	0.79 0.59-1.1	0.43 0.32-0.57	0.71 0.49-1.0	0.66 0.46-0.92	0.61 0.47-0.78	0.43 0.16-0.88
III(e) Other specified intracranial/intraspinal neoplasms	16.6 14.7-18.7	15.84 11.3-21.4	16.0 15.5-16.5	15.6 14.2-17.1	12.2 11.5-12.9	8.4 5.8-11.2	13.9 13.4-14.5	12.3 11.3-13.4	11.6 10.9-12.4	11.4 10.7-12.0	12.5 11.6-13.5	14.6 13.5-15.8	10.9 10.3-11.5	10.0 8.5-11.6
III(e.1) Pituitary adenomas and carcinomas	5.2 4.1-6.4	4.2 2.0-7.4	4.0 3.8-4.3	5.0 4.4-5.8	2.9 2.5-3.2	2.2 1.2-3.8	3.6 3.3-3.8	3.8 3.3-4.3	3.4 2.9-3.8	3.9 3.5-4.3	3.2 2.7-3.7	3.3 2.8-3.9	2.3 2.1-2.6	3.0 2.3-3.8
III(e.5) Meningiomas	11.4 9.9-13.1	11.6 7.2-16.4	11.5 11.1-11.9	10.2 9.0-11.5	9 8.4-9.6	6.1 3.8-9.1	9.8 9.4-10.3	8.1 7.2-9.0	7.9 7.3-8.5	7.1 6.6-7.7	8.8 8.0-9.6	10.8 9.8-11.9	8.1 7.6-8.7	6.8 5.6-8.2
III(f) Unspecified intracranial and intraspinal neoplasms	2.7 2.0-3.6	2.7 1.1-5.5	1.6 1.5-1.8	1.4 1.0-1.9	2.7 2.3-3.0	1.2 0.6-2.3	1.8 1.6-2.0	1.5 1.1-1.9	1.3 1.0-1.6	2.0 1.7-2.3	2.2 1.8-2.7	2.2 1.7-2.6	1.6 1.4-1.9	1.1 0.71-1.7

When grouped by gender, women had higher average annual age-adjusted incidences than men, regardless of race or geographic region.

**Figure 3:**
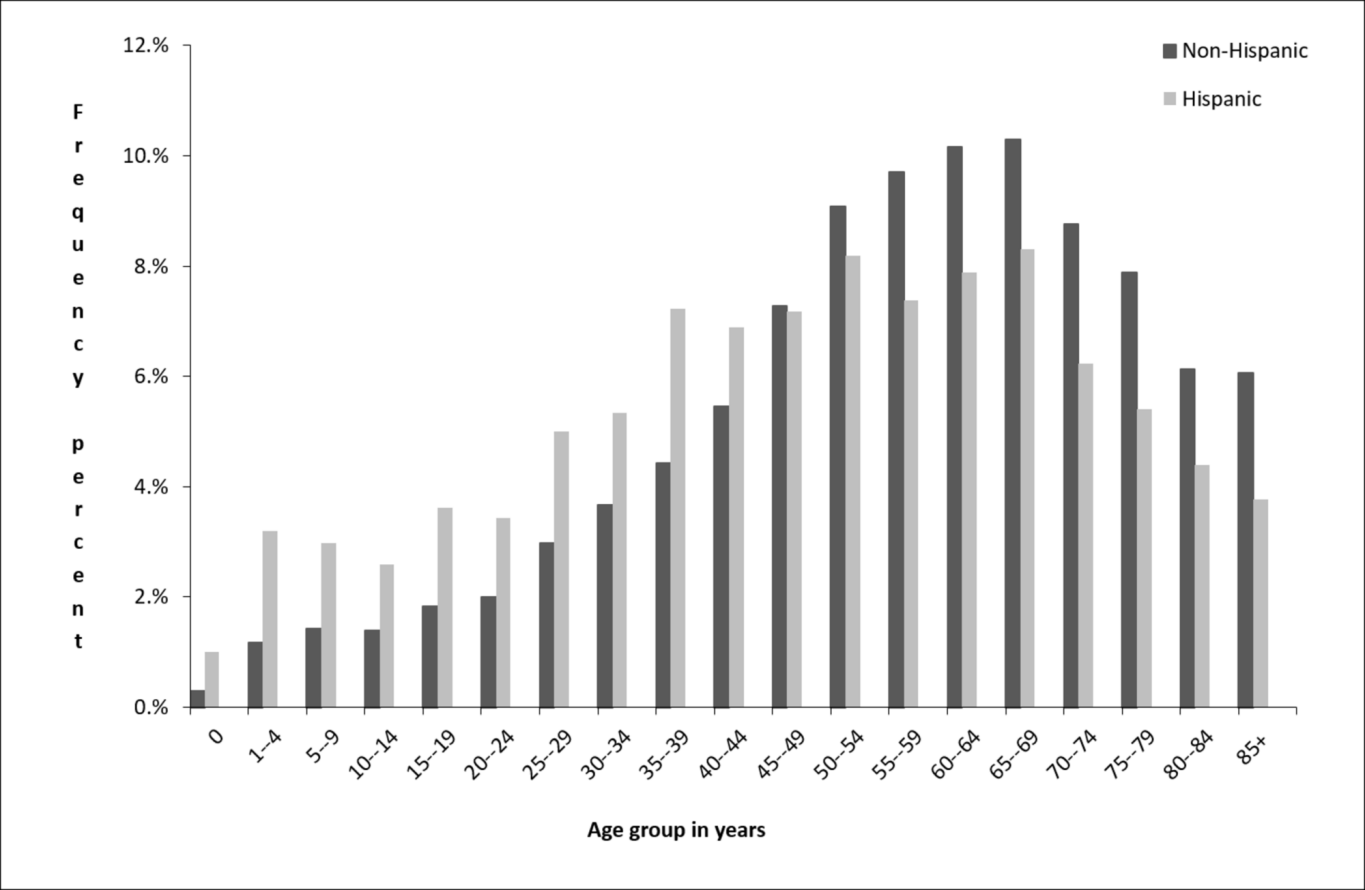
Proportions of primary CNS tumors by age group among Hispanics in Texas (2008-2012). CNS: central nervous system

There was an ethnic variation of the proportions of primary CNS tumors across age groups. Of the total count of tumors among Hispanic people, higher proportions are seen in the younger age groups (0-44 years of age), while in non-Hispanics higher proportions are seen in older age groups (45-85+ years of age). Among Hispanic populations, about half (43.3%) of all the primary CNS tumors (2008 to 2012) occurred in the age groups of 20 to 54 years while the proportion for non-Hispanics was one-third (34.9%). The highest frequency of tumors was observed in the age group of 65-69 years for both Hispanics and non-Hispanics (Figure 3).

**Figure 4:**
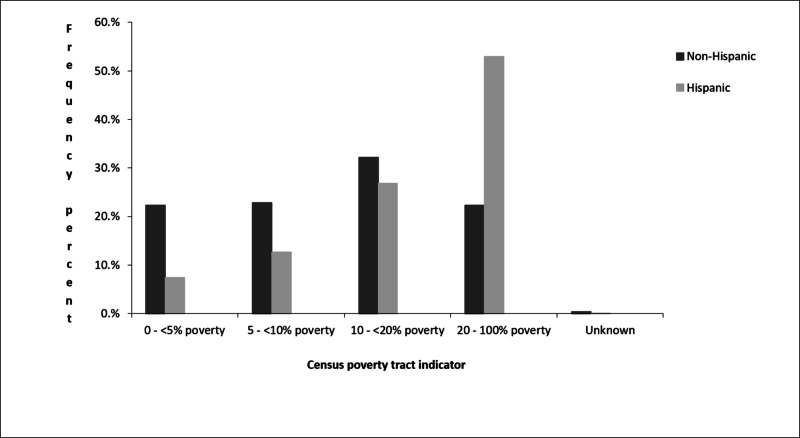
Frequency of primary CNS tumors by poverty level among Hispanics in Texas (2008 to 2012). CNS: central nervous system

With regard to socio-economic status, 53% of Hispanics with primary CNS tumors from 2008 to 2012 are within 20-100% of the poverty level, whereas 22.37% of non-Hispanics were within the same poverty level: i.e Hispanic patients were 2.4 times more than non-Hispanic patients at this high level of poverty (Figure 4).

## Discussion

Overall, the study revealed that there was a more noticeable decreasing trend of primary CNS tumor incidence among Hispanics compared to non-Hispanics. Compared with earlier studies (1985 to 1995), the decrease in incidence among Hispanics did not follow the increasing trend on the national level [3]. The average annual age-adjusted incidence (2008 to 2012) of primary CNS tumors among Hispanics and non-Hispanics combined was 25.35 per 100,000 persons with a standard error of 0.15 (95% CI: 25.06, 25.64). This is similar to the CBTRUS incidence rate for Texas from 2008 to 2012 at 24.64 (95% CI: 24.36, 24.93). A total of 30,122 cases of primary CNS tumors were diagnosed in Texas during the study period. On the national level, between 2008 and 2012, the average incidence of CNS tumors was 20.45 per 100,000 persons among Hispanics, and 22.31 per 100,000 persons among non-Hispanics [8]. This incidence trend of malignant primary CNS tumors among non-Hispanics is consistently higher than that of Hispanics for the past two decades (Figure 1). Non-Hispanics had an increasing trend until 2002 at a rate of 1.14% per year when there was a significant change in trend which started declining. However, during the same period, the Hispanic population trend continued to decline at 0.83% per year (Figure 1). Although the average incidence rate in Texas has increased for the non-Hispanic majority from 1995 to 2002; however, the overall incidence rate in the state is lower when one considers the national trend-regardless of the study period.

Women displayed predominance in incidence across the Hispanic and non-Hispanic populations and across regions of Texas. This female predominance is consistent with national statistics (CBTRUS); however, it is different from the worldwide statistics, which report a higher incidence of primary CNS tumors in men [4]. This difference could be explained by improper following of international tumors’ classification, which is not the case in the US. Other explanation could be a difference in reports focused on malignant brain tumors, thus in the US males have a predominance in malignant brain tumors compared with females. The overall incidence rates of all cancer decline at 1.8% annually among men (2001-2005), and much slower for women at 0.6% (1998-2005). Similarly, death rates among Hispanic men decreased on average by 2.3% per year compared with 1.4% per year among Hispanic women [9].

It is possible that this may be due to a few factors: genetic predisposition, diet, improved and early diagnosis, appropriate treatments, or increased healthcare coverage among the Hispanic population.

In the state of Texas, the non-Hispanic population was more likely to have malignant tumors compared with Hispanics (8.06 vs 5.93 per 100,000 age-adjusted to the 2000 US standard population). We observed notable regional differences in the average annual age-adjusted incidence rates. The highest rates were in the Panhandle for non-Hispanics (30.83) and in West Texas for Hispanics (27.17).

Even though cancer is the primary cause of mortality among Hispanics, and Texas is home to 19% of the Hispanic population living in the US, Texas and California alone comprise more than 80% of Mexican-Americans [9]. The annual percentage change among the Hispanic population is constantly decreasing (-0.83%) and increasing for non-Hispanics (1.14%) up to 2002. It should be noted that the trend curve of non-Hispanics is consistently above that of Hispanics throughout the study period.

Multiple studies have shown that risk factors for brain cancers could be related to occupational and environmental factors. Some of those risk factors are electromagnetic radiation from wireless devices, synthetic rubber manufacturing, petrochemical industries, farming, air pollutants, dietary, tobacco, and residues near landfills [10-12]. With its multiple waste landfills, many consider Texas to be the world capital for the oil and natural gas industry. The world’s largest natural gas reservoir is in the Panhandle region; shale, oil, and gas can be found in North Texas and in the West Texas region [13,14]. Extraction of natural gas has led to monumental waste that quickly exhausted the field in a short period; this was the case in the Panhandle region. These factors may have contributed to the higher incidence rates in the three regions (Figure 2). Additionally, the Hispanic mid-age groups are heavily involved in labor work and low-level education jobs like construction and have been reported to have the highest occupational health fatalities [15]. Such affiliation could explain the higher incidence among such age-groups. Additionally, low socioeconomic status, poor access to healthcare, migration, higher population concentration, and seasonal farmworker status may have contributed to the higher incidence rate in these regions among Hispanic middle age groups that have more exposure to the aforementioned contributing factors [13,16-18]. Poverty is strongly associated with higher incidence among the Hispanic population, with about 53% of patients at 20-100% poverty (Figure 4).

Although the south region has a high Hispanic population, the incidence rates are not as elevated. This could be due to less intense oil and gas activities [19]. However, despite the multiple petrochemical and other industrial and farming activities in the upper gulf, the incidence rates are lower among the Hispanic population there (21.54) but higher for non-Hispanics; this could be due to difference in industrial occupation between Hispanics and non-Hispanics in this specific region. The Central and East Texas regions have the least incidence rates and the smallest Hispanic population (Figure 2).

Even though the Joinpoint regression model is a robust model used by the National Cancer Institute (USA) to analyze cancer incidence and mortality trends, it uses the Monte Carlo Permutation method to test for significance. Like any other regression method, the Joinpoint regression method can be influenced by outliers of which our dataset had no outlying value.

The regional variation among Hispanics is likely to be a true variation because the study population is one ethnic group and, therefore, less likely to be influenced by access to health care. Furthermore, discrepancies in diagnostic and reporting practices are less likely, especially given that the diagnostic and reporting are standardized within the state of Texas. Different exposures affect different types of primary CNS tumors; therefore, it is difficult to give one explanation for the geographic variation observed, since we are considering all primary CNS tumors (for example, some epidemiologic studies have demonstrated an inverse relationship between allergies and gliomas, but no other types of brain tumors) [19].

## Conclusions

The incidence trend of malignant primary CNS tumors among Hispanics in Texas has occurred in younger age groups and declined over the past two decades (1995-2013). Generally, the incidence trend is the same in every geographic region except for Central Texas, with a rapidly declining trend. The West Texas region had the highest average age-adjusted incidence rate, followed by North Texas and the Panhandle region, while East Texas had the lowest. Females have a higher average age-adjusted annual incidence rates of primary CNS tumors and males have higher incidence rates of malignant tumors, which is consistent with national statistics. The incidence of primary CNS tumors was more pronounced in northern Texas than in southern Texas. Additionally, incidence trends and rates for primary CNS tumors demonstrated ethnic, gender, age, and socio-economic discrepancies.

These observed variations are remarkable findings because of standardized medical practice in the state of Texas. This helps to understand the distribution of disease and the necessity to create and mobilize resources towards communities for improvement or monitoring of the demographics and social state in communities. The presence of oil and gas production, farming, and construction industries might play a role in terms of occupational health leading to rate of incidence in the regions and among the younger workforce due to chronic exposure. Further prospective studies are necessary to focus on short term changes, as well as occupational and environmental determinants of health.
